# Spectrum of Myelitis in Systemic Lupus Erythematosus: Experience from a Single Tertiary Care Centre over 25 Years

**DOI:** 10.31138/mjr.32.1.31

**Published:** 2021-03-31

**Authors:** Pankti Mehta, Latika Gupta, Hafis Muhammed, Durga P. Misra, Able Lawrence, Vikas Agarwal, Amita Aggarwal, Ramnath Misra

**Affiliations:** Department of Clinical Immunology and Rheumatology, SGPGIMS, Lucknow, India

**Keywords:** SLE, lupus, myelitis, demyelinating syndromes, neuromyelitis optica spectrum disorders, rituximab, neuropsychiatric lupus

## Abstract

**Background:**

Inflammatory myelitis rarely occurs in Systemic Lupus Erythematosus (SLE).

**Methods:**

Medical records from a tertiary care centre in India (1989–2018) were reviewed to identify patients with myelitis in SLE and their clinical characteristics and outcomes were compared with two matching comparators drawn from adjacent hospital registration numbers in the SLE database.

**Results:**

Ten patients had myelitis from a cohort of 1768 patients with SLE. Myelitis was the first manifestation of lupus in 7 (70%). Cervicothoracic cord was most frequent site of involvement. ANA was negative at onset in 2 cases. One of 4 was positive for Anti-Aquaporin 4 antibody. Four had relapsing disease (16 events) with a median time to relapse of 0.65 years (0.3– 7 years). All cases received steroid sparing agents over the follow-up duration (78.5 patient years). Lupus nephritis (20% vs. 75%, p=0.004) and haematologic manifestations (0 vs. 25%, p=0.02) were less common. Higher frequency of anti-Ro antibodies was noted in the group with myelitis (p=0.05).

**Conclusion:**

Myelitis can be a presenting feature of SLE with lupus nephritis and hematologic involvement being rare. Relapses are common that mandate long-term immunosuppression.

## INTRODUCTION

Neuropsychiatric involvement in Systemic Lupus Erythematosus (NPSLE) is a major cause of morbidity and mortality. Of the 19 distinct syndromes described under NPSLE, one is myelopathy.^1,2^ Cord involvement can be attributed to demyelination, thrombosis and vasculitis specifically in SLE in addition to infective and compressive causes. Demyelinating syndromes (DS) in SLE have been previously attempted to be classified into Neuromyelitis Optica (NMO), Neuromyelitis Optica spectrum disorder (NMOSD), DS predominantly involving the brain, DS predominantly involving the brainstem and Clinically Isolated Syndrome (CIS).^3^ The rare nature of demyelinating cord disease (<1%) along with its heterogeneity^4^ has precluded a clear understating of the pathogenesis, prevalence, and clinical course in the setting of SLE. Consensus on management assumes an important role due to its potentially devastating nature, with adverse effects on the quality of life.^4,5^ Data from this part of the world is lacking with respect to this regard and thus, we conducted a retrospective chart review of a large cohort of SLE with a focus on the prevalence, clinical features, and laboratory profile of myelitis in SLE and compared those with patients without myelitis.

## METHODS

**Figure 1.**
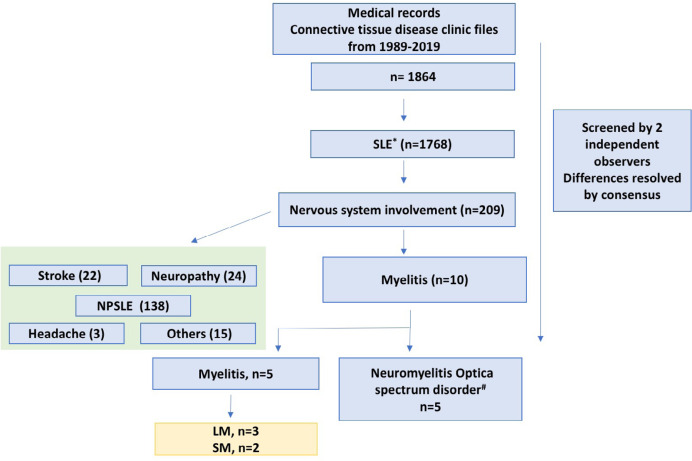
Methodology. *SLICC criteria; #by 2015 International Consensus Diagnostic Criteria for NMOSD; LM: Longitudinal myelitis; NMOSD: Neuromyelitis Optica Spectrum Disorder; NPSLE: Neuro-Psychiatric Systemic Lupus Erythematosus; SM: Short Segment Myelitis; SLICC: Systemic Lupus International Collaborating Clinic criteria; SLE: Systemic Lupus Erythematosus

A retrospective chart review was conducted to screen the records of patients with Connective tissue disease (CTD) were screened to identify SLE (SLICC criteria, **Figure 1**).^6^ Among cases with NPSLE, ^1^ myelitis were identified by the Transverse Myelitis Working Group criteria.^7^ Their demographic details, clinical profile, laboratory markers (haemogram, clinical chemistry, cerebrospinal fluid profile, autoantibodies, inflammatory markers and complements), imaging, treatment history and outcomes were recorded till the last hospital visit. A waiver of consent was taken from the Institutional review board for retrospective review of records.

Nephritis was defined as nephrotic range proteinuria or proteinuria >500mg/24 hours with active sediments with or without renal biopsy. Hematologic manifestation was defined as presence of leukopenia (<4000/cmm) and/or thrombocytopenia (<100000/cmm) and/or autoimmune haemolytic anaemia (haemolytic anaemia with Coombs positivity). Autoantibodies included Anti-Nuclear Antibodies (ANA, by Indirect Immunofluorescence), anti-double stranded DNA antibody (anti-dsDNA, by ELISA), Extractable Nuclear Antigen (by Immunoblot-Anti Smith, Ribonucleoprotein, SS-A, SS-B, P0) and anti-phospholipid antibodies (lupus anticoagulant [LAC], anticardiolipin [aCL] IgM and IgG antibodies, and anti-beta-2 glycoprotein-I [anti-β2GPI] IgM and IgG antibodies). The Systemic Lupus Erythematosus Disease Activity Index (SLEDAI)^8^ was measured retrospectively at the diagnosis of myelitis.

Longitudinal Myelitis (LM) was defined as T2 enhancement on spinal magnetic resonance imaging (MRI) of three contiguous vertebral segments and the rest labelled as short segment Myelitis (SM).^9^ The presence of Optic Neuritis (ON) was based on MRI or Visual Evoked Potentials (VEP). Relapse of either ON or myelitis was defined as new-onset neurologic impairment supported by MRI or cerebrospinal fluid (CSF) analysis, when available. Cases were classified into NMOSD when they satisfied the 2015 International Consensus Diagnostic criteria.^10^ Clinical outcomes were defined by Expanded Disability Status Scale,^11^ measured at nadir and at the time of final follow-up assessment.

For each case, two matching comparators were drawn from the previous and next hospital registration number in the lupus database. All parameters were compared with SLE patients without myelitis. Values are expressed as median and interquartile range. Categorical variables were compared using chi-square and continuous variables using Mann Whitney test. p<0.05 was taken as statistically significant. All statistics were done using SPSS (v23, IBM 2010).

## RESULTS

Of the 1864 records with CTD, 1768 were classified as SLE (**Figure 1**). Among these, 209 (11.8 %) had NPSLE, and 10 (0.56%) myelitis. All 10 were women of age 22 (16.25–24.5) and 22.5 (16.75–32.5) at the time of myelitis and diagnosis of lupus respectively. Myelitis was diagnosed concurrence with SLE in 5, and was the initial presentation in 2. The latter 2 were diagnosed as SLE 6 and 9 years after myelitis respectively.

**Figure 2.**
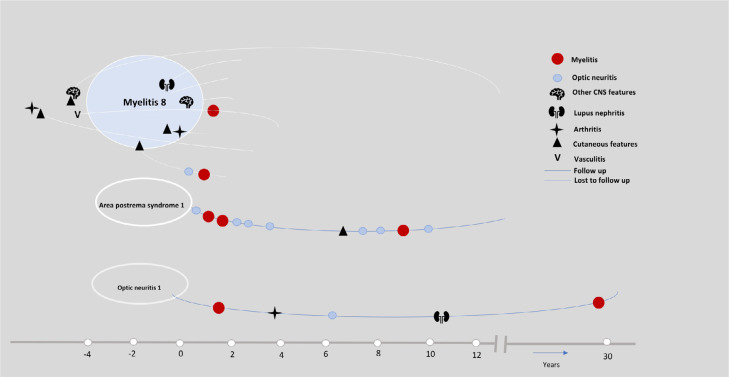
Clinical course of the patients. We have classified the patients with myelitis based on the symptom complex at presentation and depicted their clinical course over their follow up period.

While most (8, 80%) had myelitis as the first demyelinating event, 1 each had optic neuritis and area postrema syndrome to begin with, and developed myelitis during later relapses (**Figure 2**). Most patients had LM (6 of 8, 75%) at presentation. Thoracic cord (7 of 14, 50%) was the most common site, followed by cervical (4 of 14, 28%) (**Supplementary Table 2**). Notably, 5 of 10 had other incidental abnormalities on MRI head such as T2 hyperintensities in crux cerebri, basal ganglia, and centrum semiovale; lacunar infarcts in frontoparietal region and cerebellar infarct.

**Supplementary Table 2.** Investigations and treatment details of patients with myelitis.

	**Case** **1**	**Case** **2**	**Case** **3**	**Case** **4**	**Case** **5**	**Case** **6**	**Case** **7**	**Case** **8**	**Case** **9**	**Case** **10**
**Autoantibodies**										
ANA at 1:100, IIF	4+ (H)	x, 4+ (CS)	x, 4+ (FS)	(CS) 4+	(H) 4+	(CS) 4+	(H) 4+	(CS) 4+	(FS) 4+	(CS) 4+
dsDNA > 30 IU, ELISA	x	x	x	x	√	x	√	√	√	√
ENA	negative	SSA, Sm, RNP	N/A	Sm, P0	dsDNA SSA,	Sm, RNP	Sm, RNP, SSA,SSB	P0	histone, dsDNA, P0	RNP, SSA, SSB
Low complements	x	x	√	√	√	√	x	√	√	√
APS profile	x	x	√	√	x	x	x	√	√	x
**MRI**										
LM	√	√	√	x	√	√	√	√	x	√
Other	T2 HI in B/L centum semiovale	x	T2 HI in medulla	T2 HI in Basal ganglia	x	x	FP lacunar infarcts	T2 HI in R crux cerebri	R cerebellar infarct	x
**CSF**										
Elevated CSF protein	√	√	x	x	-	√	√	√	x	x
CSF pleocytosis	√	x	x	x	-	x	√	x	x	x
**Treatment**										
Steroids, =/− Pulse MPS	√	√	√	√	√	√	√	√	√	√
Cyclophosphamide	√ (NIH)	√(ELNT) √(NIH)	-	-	√(NIH)	-	√(NIH)	√(NIH)	√(NIH)	-
Azathioprine	M	-	-	√	√(M)	-	√(M)	√ (M)	√ (M)	-
Rituximab	√	√	-	-	-	-	-	-	-	√
**Outcome**										
EDSS at last follow up	1	5	LFU	2	2	LFU	1	1	1	2

ANA: Anti-Nuclear Antibodies; H: Homogenous; CS: Coarse Speckled; FS: Fine speckled; dsDNA: Double stranded deoxy ribonucleoprotein; ENA: Extractable Nuclear antigens; SSA: Anti Ro; Sm: Anti Smith Antibody; P0: Anti ribosomal P; APS: Antiphospholipid antibody syndrome profile; MRI: Magnetic resonance Imaging; LM: Longitudinal Myelitis; HI: Hyperintensities; CSF: Cerebrospinal fluid; ELNT: Euro: Lupus Nephritis Trial; NIH: National Institute of Health protocol; M: Maintenance; EDSS: Expanded Disability Status Scale; LFU: Lost to follow up

CSF examination (9 of 10) showed elevated protein in 5 (55.5%) and lymphocytic pleocytosis in 2 (22.2%).

Other features of SLE were seen in half of the cases at presentation (**Supplementary Table 1**) and notably, haematological involvement in none. ANA was negative at presentation in two, and later turned positive at 2 and 5 years respectively. Anti-Ro antibody, anti-Sm/RNP and elevated anti-dsDNA were seen in 4, 4 and 5 respectively. Four had positive antiphospholipid antibodies. NMOIgG was tested in 4 and found positive in 1 (25%). In comparison with other patients of SLE without myelitis, Ro positivity was higher in those with myelitis (p=0.05, OR 6 [0.9–41.44]). Lupus nephritis and haematological involvement were less common in patients with myelitis in lupus (p=0.004, OR 0.08 [0.01–0.53] and 0.02, OR 0.54 [0.37–0.79] respectively).

**Supplementary Table 1.** Demographic and clinical characteristics of patients with myelitis.

	**Case** **1**	**Case** **2**	**Case** **3**	**Case** **4**	**Case** **5**	**Case** **6**	**Case** **7**	**Case** **8**	**Case** **9**	**Case** **10**
**Demographics and Clinical picture**										
Age at 1^**st**^ presentation of myelitis/ON (years)	24	7	11	18	26	18	21	24	38	23
Age at diagnosis of SLE (years)	35	13	11	18	23	18	17	24	38	16
**CNS involvement (ever)**										
Paraparesis/Quadriparesis (no. of episodes)	√ (2)	√ (5)	√	√	√ (2)	√ (3)	√	√	√	√
Sensory involvement, level whenever known	√ C5, C5	√ T10	√ T7	√ C4	√ C8, L1	√ C2, T2	√ T10	x	√	√ T10
Bladder/Bowel involvement	x	√	√	√	√	√	√	x	x	√
Brain stem involvement	x	√ (Area Postrema)	x	x	x	x	x	x	x	x
Nadir EDSS1.5888.5888.5679 Associated ON, (No. of episodes)	√ (2)	Yes (5)	x	x	x	√	x	√	x	x
Other CNS features	x	x	x	ACS	x	x	CVA, S	x	X	x
**Features of lupus**										
Features of active disease at the time of myelitis	x	x	C, A	V	V	x	C	x	LN	C, A
Organ system involvement due to lupus at any point in time	LN, A	C, A	C, A	x	V	C	C, A	x	x	C, A
SELENA SLEDAI at the time of myelitis 1^st^ episode	-	-	8	18	13	8	2	12	16	4
Relapses of CNS diseases	3	10	LFU	0	1	2, LFU	0	0	0	0
Follow up duration (years)	30.6	16	1.9	2	1	0.16	13	2	1.2	7.16

ON: Optic Neuritis; SLE: Systemic Lupus Erythematosus; CNS: Central Nervous System; ACS: Acute Confusional state; CVA: Cerebrovascular Accident; S: Seizures; C: Cutaneous; A: Arthritis; LN: Lupus nephritis; V: Vasculitis; LFU: Lost to follow up

### Myelitis in lupus was prone to relapses

Sixteen episodes of relapse occurred in 4 of the 10 (40%) women over 78.5 patient-years, with a median time to relapse of 0.65 (0.3–7) years. Relapses were constituted by myelitis,^9^ ON,^6^ and 1 with features of both simultaneously. High dose glucocorticoids and cyclophosphamide (CYC) were the choice of treatment in the majority with Rituximab preferred in those with relapsing disease (**Supplementary Table 2**).

Of the 8 patients on follow up, 90% had no to minimal disability at their last follow up. The one suffering from significant disability had multiple relapses when treated with steroid monotherapy and there was a considerable delay in initiating definitive steroid sparing drug (**Supplementary Table 2**).

## DISCUSSION

Myelitis seldom occurs in SLE although is 1000 times more common than the general population.^12^ We found a prevalence of 0.56% in 1768 patients of lupus similar to other recent studies.^13^ They had presentations with motor, sensory and/or bladder bowel involvement in varying combinations with most common site of involvement being the cervicothoracic cord. A significant proportion (40%) had a relapsing course reiterating the importance of maintenance immunosuppression.

Myelitis can be the first manifestation of SLE as seen in 7 patients (70%) in our case series, even before disease manifests elsewhere which was also observed in other series.^14^ These patients were significantly less likely to have nephritis and haematologic manifestations making the diagnosis particularly challenging.^14^ Since patient with such features are likely to present to the neurologist it is important to suspect and rule out underlying autoimmune disease.

At times autoantibody positivity can be the only sign of underlying SLE which may evolve into clinical lupus with time as seen in 2 of our patients. It is pertinent to note that in this series, ANA was negative at the outset in 2 cases and the autoantibody positivity evolved with time. It is now well known that autoimmunity precedes clinical disease. Cohorts of pre-lupus have very well described the evolution of autoantibodies from anti-Ro to anti-ds DNA and later anti-Sm due to epitope spreading. Along with clinical features, the antibody profile can also evolve with time. Thus, it is important to observe these individuals over time, as manifestations of another organ involvement may appear later. Anti Ro antibody was more commonly seen in those with myelitis as seen in other cohorts.^15^ Antiphospholipid antibody is also commonly reported in patients with myelitis (40% in our series) and it may be associated with LM, however none of them had other clinical features of antiphospholipid antibody syndrome.^4^

CSF analysis revealed elevated protein in 5 of 9 and lymphocytosis in 2 of 9. CSF in NMOSD is characterized by lymphocytic pleocytosis, however, it was not observed in our cohort. Prevalence of CSF pleocytosis varies widely in NMOSD, ranging from 14 to 79 percent.^16,17^ In addition, CSF changes are dynamic and often related to disease activity. False negatives for CSF pleocytosis can also result from very early CSF examination, erroneous sample handling or delayed processing. Probably, one of these could be the reason for the discrepancy.

Further, we found that 5 (50%) of our patients could be classified into NMOSD.^10^ NMOSD overlap can occur in patients with other autoimmune diseases, the most common being SLE. Data on myelitis in the setting of other rheumatic disorders has been detailed in **Supplementary Table 3**.^13,18,19^ The other series shows female preponderance, although patients were younger in our series. LM was seen in most cases, but ON was higher in the case series from Tianjin. This could be due to routine Visual Analogue Potential testing in that cohort. Petri et al. have classified cord involvement into white matter and gray matter disease.^4^ It is important to note that although all cases improved with high dose glucocorticoids, a large proportion relapsed on follow-up, highlighting the need for steroid sparing immunosuppressants.^14^

**Supplementary Table 3.** Comparison with case series of NMO SD associated with CTD.

	**SGPGI, Lucknow, India**	**University of Colorado, Denver, USA (2011)19**	**University Gen Hospital, Tianjin, China (2018)29**	**Hospital ‘Carlos G Durand’, Buenos Aires, Argentina (2016)18**	**Harvard Medical School, Boston, USA (2019)13**
Lupus cohort	**1768**	**-**	**-**	233	2297
No. of patients with lupus myelitis	**10 (0.56%)**	13	18	5	15 (0.7%)
Mean Age (years)	**20.33**	38.3	39	25.4	
Gender- F:M	**10:0**	11:2	18:0	5:0	13:2
Type of CTD • Lupus • Primary Sjogren’s syndrome • Lupus with secondary Sjogren’s syndrome • Lupus with APS	**10**	2 3 1 -	3 7 - -, RA-1, UCTD-7	5	15
LM	**8 (80%)**	6 (46%)	15 (83%)	3 (60%)	4 (26.67%)
ON	**5 (50%)**	6 (46%)	14 (77%)	-	4 (26.67%)
Anti AQP4 Ab • Lupus • Primary Sjogren’s syndrome • Lupus with Secondary Sjogren’s syndrome	**1 (25%) (tested in 4)**	0 3 (100%) 1 (100%)	12 (77%)	0/2 tested	-
Treatment (Induction) • Glucocorticoids • CYC • RTX • AZA • PLEX • MMF	**10 (33% failed)** **3 (remission)** **1 (remission)** **-** **-**	4 2 - - 1 -	-	5 3 - - 1 -	13 2 1 1 2 2
Treatment (Maintenance) • RTX • AZA/MMF	**3** **6 (1 relapse, shifted to Rituximab)**	- 3	-	-	-
Outcome Response to therapy Relapse	**EDSS (1–5)** **16 relapses in 4 patients;** **Steroids (n=4), 13 relapses** **Cyclophosphamide, (n=2), 3 relapses**	- Steroid monotherapy(n=4)-mod/severe functional impairment, 19 relapses Maintenance therapy with AZA/MMF- minimal functional impairment, 2 relapses	- -	AIS A, B or C at 6 months	AIS category D or E at 1 year follow up

CTD: Connective Tissue Disease, anti AQP4 Ab: Anti Aquaporin 4 antibodies, APS: Anti Phospholipid antibody Syndrome, LM: Longitudinally Extensive Transverse Myelitis, ON: Optic Neuritis, AZA: Azathioprine, CYC: Cyclophosphamide, RTX: Rituximab, PLEX: Plasma exchange, MMF: Mycophenolate Mofetil, AIS: American Spinal Injury Association Impairment Scale, EDSS: Expanded Disability Status Scale

Myelitis is treated by various drugs and regimens with an induction and maintenance phase. Induction can be done with pulse methylprednisolone (1000mg intravenously for 3–5 days); however, if the initial response is inadequate then plasmapheresis may be considered.^20,21^ This must be followed by a maintenance regimen as the main aim of therapy is to prevent subsequent relapses, prevent steroid toxicity and additive disability. Maintenance regimens may consist of Azathioprine, MMF, cyclophosphamide, or Rituximab.^22,23,24^

In our case series, while one-thirds of those receiving CYC relapsed, all of the 3 on RTX did well on follow-up. This is also in line with previous findings in a metanalysis by Fulin Gao et al. wherein 46 studies involving 438 patients on rituximab therapy, resulted in a mean (SE) 0.79 (0.15) reduction in the mean annualized relapse rate ratio reduction in the Disability Status Scale score.^25^ Thus, RTX is emerging as first line steroid sparing agent in NMO.^23^ It seems likely that it would work as well in NMO-SD too. Monthly CD 27 monitoring can identify short term responders to RTX as described by Ciron et al. in recommendations for the use of Rituximab in NMOSD.^26^ Recent reports suggest successful use of Eculizumab (monoclonal antibody against C5a) and Inebilizumab (previously known as MEDI-551), a CD19 monoclonal antibody in NMOSD.^27,28^

This case series fills important lacunae in existent knowledge of myelitis in the setting of lupus, and offers novel insights for the rheumatologist and neurologist alike. It also suggests that evolution of disease and autoantibody profile could vary, and a high index of suspicion for lupus adds greatly to diagnosis and successful therapy. The study has the disadvantages inherent to any retrospective analysis. Further, anti-aquaporin antibody was not tested in all cases. Also, cases were recruited from the rheumatology department, and possibly cases presenting to neurology department could have been missed.

Thus, demyelinating syndromes can be the first manifestation of lupus. Although NMOSD is the most common form, CIS can also be seen in setting of lupus. Lupus nephritis and hematologic manifestations are less common, and ANA can be negative in lupus with myelitis making the diagnosis challenging at times. The disease should be aggressively treated with cyclophosphamide or rituximab followed by maintenance immunosuppression to prevent disability.

## CODE AVAILABILITY

Custom Coding.
